# Implementierung eines nationalen Lungenkarzinom-Screening in Österreich: Positionspapier der Österreichischen Röntgengesellschaft (ÖRG), der Österreichischen Gesellschaft für Pneumologie (ÖGP), der Österreichischen Gesellschaft für Allgemeinmedizin und Familienmedizin (ÖGAM) und der Österreichischen Gesellschaft für Thoraxchirurgie (ÖGTC)

**DOI:** 10.1007/s00508-026-02813-w

**Published:** 2026-09-18

**Authors:** Romana Wass, Gerlig Widmann, Herbert Bachler, Thomas Klikovits, Judith Löffler-Ragg, Martin Uggowitzer, Peter Kowatsch, Florian Augustin, Arschang Valipour, Klaus Wicke, Florian Tomaselli, Marie-Kathrin Breyer, Stefan Watzka, Maximilian Hochmair, Eveline Kink, Marcel Rowhani, Bernd Lamprecht, Helmut Prosch

**Affiliations:** 1 Universitätsklinik für Innere Medizin 4 - Pneumologie, Medizinische Fakultät, Johannes Kepler Universität Linz, Altenberger Straße 69, 4040 Linz, Österreich; 2 Klinik für Radiologie, Medizinische Universität Innsbruck, Innsbruck, Österreich; 3 Österreichische Gesellschaft für Allgemeinmedizin und Familienmedizin, Innsbruck, Österreich; 4 Abteilung Thoraxchirurgie, Wiener Gesundheitsverbund, Klinik Floridsdorf Wien, Wien, Österreich; 5 Karl Landsteiner Institut für thoraxchirurgische Forschung, Klinik Floridsdorf Wien, Wien, Österreich; 6 Abteilung Pneumologie, Landeskrankenhaus Hochzirl-Natters, Natters, Österreich; 7 Innere Medizin II, Medizinische Universität Innsbruck, Innsbruck, Österreich; 8 Institut für Radiologie und Nuklearmedizin, Landeskrankenhaus Hochsteiermark, Leoben, Österreich; 9 Österreichische Gesellschaft für Allgemein- & Familienmedizin, Sankt Gilgen, Österreich; 10 Universitätsklinik für Visceral‑, Transplantations- und Thoraxchirurgie, Medizinische Universität Innsbruck, Innsbruck, Österreich; 11 Abteilung für Innere Medizin und Pneumologie, Wiener Gesundheitsverbund, Klinik Floridsdorf Wien, Wien, Österreich; 12 Institut für Lungenforschung und pneumologische Onkologie, Karl Landsteiner Gesellschaft, St. Pölten, Österreich; 13 Bundesfachgruppe Radiologie der österreichischen Ärztekammer, Wien, Österreich; 14 Universitätsklinik für Herz-, Gefäß- und Thoraxchirurgie, Medizinische Fakultät, Johannes Kepler Universität, Linz, Österreich; 15 Ludwig Boltzmann Institut für Lungengesundheit, Wien, Österreich; 16 Abteilung für Atemwegs- und Lungenerkrankungen, Klinik Ottakring, Wien, Österreich; 17 Medizinische Fakultät, Sigmund Freud PrivatUniversität, Wien, Österreich; 18 Paracelsus Medizinische Privatuniversität, Salzburg, Österreich; 19 Abteilung für Innere Medizin und Pneumologie, Landeskrankenhaus Graz II, Graz, Österreich; 20 Bundesfachgruppe Lungenkrankheiten der österreichischen Ärztekammer, Wien, Österreich; 21 Universitätsklinik für Radiologie und Nuklearmedizin, Medizinische Universität Wien, Wien, Österreich

**Keywords:** Lungenkrebs-Screening, Niedrigdosiscomputertomographie (LDCT), Krebsfrüherkennung, Öffentliche Gesundheit, Österreich, Lung cancer screening, Low-dose computed tomography (LDCT), Early detection of cancer, Public health, Austria

## Abstract

**Hintergrund:**

Das Lungenkarzinom ist in Österreich eine der häufigsten Krebstodesursachen und wird häufig erst in fortgeschrittenen Stadien diagnostiziert. Internationale Studien belegen, dass ein strukturiertes Screening mittels Niedrigdosiscomputertomographie die lungenkrebsbedingte Sterblichkeit senkt und die Diagnose in frühere, kurativ behandelbare Stadien verlagert. Ziel dieses Positionspapiers ist es, eine evidenzbasierte Grundlage für die Einführung eines nationalen Lungenkarzinom-Screening-Programms in Österreich zu schaffen.

**Methoden:**

Grundlage des Positionspapiers waren die verfügbare internationale Evidenz, aktuelle europäische Leitlinien und Empfehlungen sowie österreichische epidemiologische und gesundheitsökonomische Daten. Vertreter:innen der Österreichischen Röntgengesellschaft, der Österreichischen Gesellschaft für Pneumologie, der Österreichischen Gesellschaft für Allgemeinmedizin und Familienmedizin sowie der Österreichischen Gesellschaft für Thoraxchirurgie erarbeiteten gemeinsame Empfehlungen zur Zielpopulation, Organisation, Qualitätssicherung und Implementierung eines nationalen Screening-Programms.

**Ergebnisse:**

Das Positionspapier definiert die Zielpopulation, Ein- und Ausschlusskriterien sowie Anforderungen an Diagnostik, Qualitätssicherung, Organisation und interdisziplinäre Versorgung eines nationalen Screening-Programms. Die beteiligten Fachgesellschaften sprechen sich gemeinsam für die Einführung eines qualitätsgesicherten, risikoadaptierten Lungenkarzinom-Screenings in Österreich aus.

**Schlussfolgerung:**

Die verfügbare Evidenz spricht für die Einführung eines nationalen, qualitätsgesicherten Lungenkarzinom-Screenings in Österreich. Ein nationales Lungenkarzinom-Screening-Programm bietet die Möglichkeit, die Lungenkrebssterblichkeit nachhaltig zu senken, mehr Tumoren in kurativ behandelbaren Stadien zu diagnostizieren und die Versorgung von Hochrisikopersonen in Österreich langfristig zu verbessern. Das vorliegende Positionspapier stellt eine wissenschaftlich fundierte Grundlage für die gesundheitspolitische Umsetzung eines nationalen Lungenkarzinom-Screenings dar.

## Abstract (en)

**Background:**

Lung cancer is one of the leading causes of cancer-related mortality in Austria and is frequently diagnosed at an advanced stage. International studies have demonstrated that structured screening using low-dose computed tomography reduces lung cancer mortality and increases the detection of early-stage, potentially curable disease. The aim of this position paper is to provide an evidence-based framework for the implementation of a national lung cancer screening program in Austria.

**Methods:**

This position paper is based on the available international evidence, current European guidelines and recommendations as well as Austrian epidemiological and health economic data. Representatives of the Austrian Radiological Society, the Austrian Society of Pneumology, the Austrian Society of General Practice and Family Medicine and the Austrian Society of Thoracic Surgery jointly developed recommendations regarding the target population, program organization, quality assurance and implementation of a national lung cancer screening program.

**Results:**

The position paper defines the target population, inclusion and exclusion criteria and the requirements for diagnostics, quality assurance, program organization and multidisciplinary care within a national screening program. The participating scientific societies jointly endorse the implementation of a quality-assured, risk-adapted national lung cancer screening program in Austria.

**Conclusion:**

The available evidence supports the implementation of a national quality-assured lung cancer screening program in Austria. Such a program has the potential to sustainably reduce lung cancer mortality, increase the detection of tumors at curable stages and improve the long-term care of individuals at high risk for lung cancer. This position paper provides a scientifically sound basis for the health policy implementation of a national lung cancer screening program in Austria.

Das Lungenkarzinom bleibt in Österreich eine der schwerwiegendsten onkologischen Herausforderungen: Es zählt zu den häufigsten und tödlichsten Tumorerkrankungen und wird vielfach erst in fortgeschrittenen, nicht mehr kurativ behandelbaren Stadien diagnostiziert. Ziel dieses Positionspapiers ist es, die medizinischen, wissenschaftlichen und gesundheitspolitischen Argumente für die Einführung eines nationalen Lungenkarzinom-Screening-Programms darzustellen und damit die Grundlage für eine evidenzbasierte Entscheidungsfindung zu schaffen.

Ein strukturiertes Screening mittels Low-Dose-CT (LDCT) ermöglicht die Detektion von Lungenkarzinomen in frühen, potenziell heilbaren Stadien. Internationale randomisierte Studien zeigen eine signifikante Senkung der lungenkarzinombedingten Mortalität und belegen, dass LDCT-Screening zu einem deutlichen Stadienshift hin zu therapierbaren Tumoren führt. Für Österreich besitzt dies besondere Relevanz: Die nationale Epidemiologie zeigt eine hohe Inzidenz, ein hohes medianes Diagnosealter und eine weiterhin unzureichende Früherkennung.

Neben einer deutlich verbesserten Prognose eines früh diagnostizierten Lungentumors erfordert ein niedriges Tumorstadium weniger invasive und belastende Therapien, wodurch auch die Lebensqualität verbessert wird.

Darüber hinaus bietet ein strukturiertes Screening-Programm Synergieeffekte für die öffentliche Gesundheit: Es ermöglicht die systematische Einbindung von Raucher:innenentwöhnung und unterstützt damit präventionsorientiertes Gesundheitsverhalten.

In Summe zeigt sich, dass ein nationales LDCT-Screening ein wirksames, evidenzbasiertes und gesundheitspolitisch sinnvolles Instrument darstellt, das sowohl die Mortalität senken als auch die Versorgungslast des Gesundheitssystems langfristig reduzieren kann.

## Hintergrund und Evidenzlage

Das Lungenkarzinom ist in Österreich die zweithäufigste Krebserkrankung sowohl bei Frauen als auch bei Männern. Im Jahr 2024 wurde bei 2513 Frauen und 3020 Männern eine Lungenkarzinomneuerkrankung diagnostiziert. Bei Männern ist das Lungenkarzinom die häufigste Krebstodesursache; rund jeder fünfte Krebssterbefall (21 %) ist durch Lungenkarzinom bedingt. Bei Frauen nimmt das Lungenkarzinom die zweite Stelle der krebsbedingten Todesursachen nach dem Mammakarzinom ein [1].

Trotz neuer therapeutischer Optionen wie zielgerichteter Therapien und Immuncheckpointinhibitoren ist die Prognose insgesamt schlecht. Das 5‑Jahres-Überleben liegt aktuell bei nur rund 20 %. Ursächlich dafür ist v. a. die häufig späte Diagnose: Bei mehr als der Hälfte der Neudiagnosen wird der Tumor erst festgestellt, wenn er bereits die Organgrenzen durchbrochen hat. Wird ein Lungenkarzinom hingegen in einem frühen, lokal begrenzten Tumorstadium diagnostiziert, kann das 5‑Jahres-Überleben deutlich auf über 50 %, im Stadium I sogar auf über 90 % angehoben werden.

Lungenkarzinom-Screening-Programme mittels LDCT können durch die Diagnose von Lungenkarzinomen in einem früheren Stadium dazu beitragen, sowohl die medizinische als auch die ökonomische Last für das Gesundheitssystem deutlich zu verringern.

Im Jahr 2010 konnte erstmals in einer prospektiven, randomisierten Studie, dem National Lung Screening Trial (NLST) in den Vereinigten Staaten, gezeigt werden, dass durch ein LDCT-Screening im Vergleich zu einem Screening mit Lungenröntgen die Lungenkarzinommortalität um 20,2 % und die Gesamtmortalität um 6,7 % gesenkt werden kann [2].

Auch die belgisch-niederländische NELSON (NEderlands-Leuvens Longkanker Screenings ONderzoek)-Studie konnte zeigen, dass die Lungenkarzinommortalität durch ein LDCT-Screening im Vergleich zu einer Kontrollgruppe ohne Screening bei Männern um 24 % gesenkt werden kann. Bei Frauen war in dieser Studie die Senkung der Mortalität mit 33 % sogar noch höher, der Unterschied war aufgrund der geringeren Fallzahl jedoch nicht statistisch signifikant [3].

Zahlreiche weitere randomisiert kontrollierte Studien, die in Italien (MILD (Multicentric Italian Lung Detection)/BioMILD, ITALUNG (Italian Lung Cancer Screening Trial), DANTE (Detection and Screening of Early Lung Cancer by Novel Imaging Technology and Molecular Essays Trial), Dänemark (Danish Lung Cancer Screening Trial/DLCST), Frankreich (French randomized pilot trial of lung cancer screening/DEPISCAN), im Vereinigten Königreich (UK Lung Cancer Screening Trial/UKLS) und in Deutschland (German Lung Cancer Screening Intervention Trial/LUSI) durchgeführt wurden, spiegeln die Ergebnisse von NLST und NELSON wider und erhärten somit klar die Evidenzlage für den positiven Nutzen eines strukturierten Lungenkarzinom-Screenings [4–10].

## Internationale Leitlinien und EU-Empfehlungen

Internationale radiologische und pneumologische Fachgesellschaften wie die European Radiology and Respiratory Society (ESR/ERS) sprechen sich vor diesem Hintergrund klar für die Umsetzung eines LDCT-Screenings zur Lungenkrebsfrüherkennung aus [11].

Internationale Leitlinien empfehlen ein strukturiertes Lungenkarzinom-Screening mittels LDCT für definierte Hochrisikogruppen:

- Die US Preventive Services Task Force (USPSTF) empfiehlt ein jährliches LDCT-Screening für Personen im Alter von 50 bis 80 Jahren mit mindestens 20 Packungsjahren Raucher:innenanamnese, die aktuell rauchen oder in den letzten 15 Jahren aufgehört haben [12].
- Die European Society for Medical Oncology (ESMO) und die European Respiratory Society (ERS) gemeinsam mit der European Society of Radiology (ESR) empfehlen LDCT-Screening für Personen im Alter von 55 bis 74 Jahren mit mindestens 30 Packungsjahren oder Ex-Raucher:innen mit weniger als 15 Jahren Abstinenz [13, 14].
- ERS und die ESR betonen darüber hinaus die Notwendigkeit multidisziplinärer Teams, Qualitätssicherung sowie der Integration von Raucher:innenentwöhnung [15].

Die EU-Kommission hat 2022 eine Ratsempfehlung ausgesprochen, die den Mitgliedstaaten empfiehlt, schrittweise nationale Lungenkarzinom-Screening-Programme für aktuelle und ehemalige starke Raucher:innen im Alter von 50 bis 75 Jahren einzuführen, um die Mortalität zu senken und den Zugang für Hochrisikogruppen zu verbessern [16, 17].

Basierend auf dieser robusten Datenlage wurde bisher in 5 Ländern Europas (Kroatien, Tschechien, Polen, Vereinigtes Königreich) ein nationales Screening-Programm etabliert. In weiteren 18 Ländern werden Programme zur Implementierung eines strukturierten Screenings vorbereitet oder pilotiert, darunter Belgien, Dänemark, Deutschland, Estland, Frankreich, Griechenland, Ungarn, Italien, Niederlande, Russland, Weißrussland, Serbien, Spanien, Schweden und die Schweiz (Liechtenstein assoziiert).

Das SOLACE (Strengthening the Screening of Lung Cancer in Europe), ein EU4Health Projekt unter dem Europe’s Beating Cancer Plan, widmet sich der Implementierung des Lungenkrebs-Screenings [18]. Ziel ist es, den gleichberechtigten Zugang zum LDCT-Screening zu verbessern, indem gezielte Rekrutierungsstrategien für unterrepräsentierte Gruppen und Hochrisikokollektive entwickelt werden. Durch umfassende Arbeitspakete integriert SOLACE wissenschaftliche Forschung, Pilotstudien und Nachhaltigkeitsbemühungen, um regionale und nationale Screening-Bemühungen in den EU-Mitgliedstaaten zu stärken [18].

## Zielgruppe und Einschlusskriterien

Die Auswahl der Teilnehmer:innen an einem Lungenkarzinom-Screening ist ein wesentlicher Faktor sowohl für die Effektivität des Screenings als auch für dessen ökonomische Auswirkungen (Kosteneffizienz). Zentral ist die Vortestwahrscheinlichkeit für ein Lungenkarzinom: Wird die Screening-Population zu großzügig definiert, sinkt die Vortestwahrscheinlichkeit, die Zahl der richtig positiven Befunde nimmt ab, und die Zahl der falsch positiven Befunde steigt. Damit steigen nicht nur die Kosten, sondern auch die Zahl der Komplikationen, die bei der Abklärung falsch positiver Befunde auftreten. Zudem fällt in einer Population mit niedriger Vortestwahrscheinlichkeit das Ausmaß der Mortalitätsreduktion deutlich geringer aus.

## Definition der Hochrisikogruppe

Hauptrisikofaktoren für das Lungenkarzinom sind das Rauchen und ein höheres Alter. Bei Raucher:innen steigt das Risiko mit der kumulativen Dauer und Menge des Zigarettenkonsums, während es bei Ex-Raucher:innen mit zunehmender Zeit seit dem Rauchstopp abnimmt.

In den meisten prospektiven Screening-Studien wurden die Einschlusskriterien über Alter und Raucher:innenanamnese definiert. Die publizierten Daten gelten dementsprechend v. a. für Personen mit langjähriger Raucher:innenanamnese und einem Alter von > 50 bis 55 Jahren. Durch Hinzunahme weiterer Kriterien kann die Risikopopulation weiter eingegrenzt und die Vortestwahrscheinlichkeit erhöht werden. Das führt zu einer höheren Rate richtig positiver LDCT-Untersuchungen und einer verbesserten Kosten/Nutzen-Relation.

PLCOm2012 ist ein validiertes Modell zur Abschätzung des Lungenkrebsrisikos, basierend auf Daten der PLCO-Studie (Prostate, Lung, Colorectal and Ovarian Cancer Screening Trial) [19, 20]. Neben Alter und Raucher:innenanamnese fließen individualisierte Variablen ein, etwa Ethnie, Bildungsniveau, Body-Mass-Index (BMI), frühere nikotinassoziierte Tumorerkrankungen, Familienanamnese von Lungenkrebs sowie chronisch obstruktiver Lungenerkrankung (COPD) und/oder Emphysem.

Erste Auswertungen der HANSE (Holistic Implementation Study Assessing a Northern German Interdisciplinary Lung Cancer Screening Effort)-Studie (Deutschland) zeigen, dass durch Verwendung des PLCOm2012-Modells im Vergleich zu den in der NELSON-Studie angewandten Einschlusskriterien etwa 20 % mehr Lungenkarzinome detektiert werden konnten [21]. PLCOm2012 zeigte gegenüber den ursprünglichen USPSTF-/NLST und NELSON Kriterien eine höhere Sensitivität (80,1 % vs. 71,2 %) und Spezifität (66,2 % vs. 62,7 %). Das später entwickelte PLCOall2014 schließt auch Nieraucher:innen ein. Programme wie das britische Targeted Lung Health Check (TLHC) und die EU-Studie 4-IN THE LUNG RUN untersuchen, wie optimierte Einschlusskriterien die Screening-Population weiter verbessern können [22, 23].

Erstmalig konnte nunmehr das erweiterte Modell, PLCO_2014all_ erfolgreich auch in Österreich in einer Pilotstudie angewandt werden. Österreichs einzige epidemiologische Langezeitgesundheitsstudie [24], LEAD (Lung, hEart, sociAl, boDy), konnte aus ihrer Kohorte von mehr als 15.000 Teilnehmer:innen jene selektieren, die als Hochrisikopopulation gelten.

## Wie viele Personen in Österreich könnten von einem Screening profitieren?

Auf Basis der österreichischen Gesundheitsstudie LEAD konnten genaue Daten zum Rauchverhalten in Österreich über die vergangenen 14 Jahre gesammelt und ausgewertet werden. Im Jahr 2024 fand sich österreichweit eine Raucher:innenprävalenz von 16,1 % bei Frauen und 20,0 % bei Männern. In der Altersgruppe der 50- bis 75-Jährigen liegt die Raucher:innenprävalenz bei Frauen bei 15,6 % und bei Männern bei 14,2 %. Da die LEAD-Studie im Bereich Alter, Geschlecht und Raucher:innenprävalenz repräsentativ für Österreich ist (Vergleich mit Statistik Austria Mikrozensus) kann somit eine sehr genaue Schätzung über die Höhe einer möglichen Screening-Population getroffen werden.

- Die Zielgruppe der 50- bis 75-Jährigen umfasst rund 2,2–2,4 Mio. Menschen.
- Unter Berücksichtigung des Alters, der Raucher:innenprävalenz, Pack-Year-Verteilung und Ex-Raucher:innenquoten erfüllen *rund 273.153 Personen in Österreich die Hochrisikokriterien *für den Einschluss in ein Lungenkrebs-Screening.

Dies deckt sich mit Modellierungen des Austrian Institute for Health Technology Assessment (AIHTA), das für Österreich eine potenzielle Screening-Population von etwa 300.000 Personen annimmt. Bei einer Teilnahmequote von 50 % wären somit rund 142.312 jährliche LDCT-Screenings zu planen. Somit würden sich rund 6019 Personen mit einem nach Kriterien der European Society of Thoracic Imaging (ESTI) abklärungsbedürftigen radiologischen Befund finden [25].

## Geschlechtsspezifische Aspekte

Besondere Beachtung sollten in Screening-Programmen auch unterrepräsentierte Populationen finden. Interessant ist, dass in mehreren Studien v. a. bei Frauen eine stärkere Senkung der lungenkrebsspezifischen Mortalität beobachtet wurde. In der NELSON-Studie lag die relative Risikoreduktion bei Frauen bei 33 % [3], wenngleich dieser Unterschied aufgrund der geringen Zahl an eingeschlossenen Frauen nicht statistisch signifikant war. In der deutschen LUSI-Studie mit rund 4000 Teilnehmer:innen und jährlichen LDCT-Untersuchungen über 4 Jahre liegt der aus diesen Daten berechnete Effekt sogar bei 69 % [10]. Auch in kleineren, nationalen Studien zeigte sich durchwegs ein stärkerer Effekt bei Frauen. Die Ursachen für diese Geschlechtsunterschiede sind noch nicht vollständig geklärt; auffällig ist jedoch, dass bei Frauen häufiger Adenokarzinome diagnostiziert werden. Die französische CASCADE-Studie (Lung CAncer SCreening in French women using low-dose CT and Artificial intelligence for DEtection) fokussiert sich aus diesem Grund auf ein Screening von Frauen [26].

## Ausschlusskriterien

Neben der Festlegung von Einschlusskriterien ist es ebenso wichtig, klare Ausschlusskriterien zu definieren. Diese sollen Personen identifizieren, die nicht von einem Screening profitieren würden – etwa, weil ein entdecktes Lungenkarzinom aufgrund schwerer Komorbiditäten nicht mehr kurativ behandelbar wäre oder weil ihre Lebenserwartung so begrenzt ist, dass ein Screening keinen relevanten gesundheitlichen Nutzen mehr erwarten lässt. Zu den Ausschlusskriterien eines nationalen risikostratifizierten Screening-Programms sollten daher unter anderem eine deutlich eingeschränkte Lebenserwartung von weniger als 5 Jahren, schwerwiegende Komorbiditäten, die eine kurative Therapie unwahrscheinlich machen, bereits bekannte oder sich in Abklärung befindliche Lungenrundherde sowie klinische Konstellationen gehören, bei denen der Einschluss keinen Benefit erwarten lässt. Diese Empfehlung basiert unter anderem auf dem Konzept des „time to benefit“, da eine Reduktion der Lungenkarzinomsterblichkeit durch Screening in der Regel erst nach mehreren Jahren nachweisbar ist. Vor diesem Hintergrund ist davon auszugehen, dass Personen mit deutlich limitierter Lebenserwartung keinen relevanten Nutzen aus einem Screening ziehen [27, 28].

Eine pragmatische und im gesundheitspolitischen Kontext handhabbare Operationalisierung dieser Empfehlung stellt die Definition einer Lebenserwartung von unter 5 Jahren dar.

## Screening-Methode und diagnostische Abläufe

Das empfohlene Verfahren zur Früherkennung des Lungenkarzinoms ist die Low-Dose-Computertomographie (LDCT) des Thorax ohne Kontrastmittel. LDCT bietet eine deutlich höhere Sensitivität für die Detektion kleiner pulmonaler Rundherde als das konventionelle Röntgen und ermöglicht gleichzeitig eine sehr niedrige Strahlenexposition.

Die von der European Society of Thoracic Imaging (ESTI) publizierten technischen Standards für das Lungenkrebs-Screening empfehlen den Einsatz von Mehrzeilen-CT-Systemen mit mindestens 32 (idealerweise ≥ 64) Detektorzeilen, einer Thoraxabdeckung vom Apex bis zur Lungenbasis, Scanzeiten von ≤ 10 s in einem Atemzug sowie einer Feld-of-View-Einstellung knapp über den Thorax hinaus, um eine hohe räumliche Auflösung und verlässliche Volumetrie zu gewährleisten [25].

Die Strahlendosis soll strikt niedrig gehalten werden. ESTI gibt CTDIvol (volumetrischer Computertomographie-Dosisindex) von etwa 0,4–1,6 mGy in Abhängigkeit vom Körpergewicht vor und empfiehlt den Einsatz von automatischer Strom- und kV-Modulation sowie iterativen oder Deep-Learning-Rekonstruktionsverfahren. Moderne LDCT-Protokolle erreichen so effektive Dosen von etwa 0,6–1,5 mSv pro Untersuchung und liegen damit unter der durchschnittlichen, jährlichen Strahlenexposition in Österreich (ca. 6 mSv [Radioaktivität und Strahlung in Österreich 2022, Bundesministerium Klimaschutz, Umwelt, Energie, Mobilität, Innovation und Technologie]).

## Untersuchungsintervall

Das Intervall des Screenings richtet sich nach dem individuellen Risikoprofil. Internationale Leitlinien sowie die großen randomisierten Studien sprechen für ein jährliches LDCT-Screening in definierten Hochrisikokollektiven, um die Detektionsrate früher Tumorstadien zu maximieren und Intervallkarzinome zu minimieren.

## Radiologische Befundung

Die Qualität und Einheitlichkeit der Befundung ist entscheidend für die Effektivität und Sicherheit des Screening-Programms. Internationale radiologische Fachgesellschaften – insbesondere ESTI und ESR – empfehlen den Einsatz standardisierter Befundungs- und Dokumentationssysteme [29].

Die ESTI-Technical Standards fordern eine Auswertung auf Workstations mit Scroll-Möglichkeit, Maximumintensitätsprojektion (MIP)/multiplanare Rekonstruktionen (MPR) sowie eine Rekonstruktion mit dünnen Schichten (≤ 1,0 mm, möglichst 0,75 mm) und standardisierten Rekonstruktionskernen, um eine verlässliche volumetrische Messung von Lungenrundherden zu ermöglichen.

Zur strukturierten Dokumentation empfiehlt ESTI ein standardisiertes LCS (Lung Cancer Screening)-Befundtemplate, in dem u. a.

- Datum der aktuellen und, falls vorhanden, vorangegangenen Untersuchung,
- Dosisangabe: CTDIvol,
- Rundherde: Typ (solid, part-solid, non-solid), Morphologie (z. B. spikuliert, perifissural), Volumen, Volumenverdopplungszeit und empfohlene weitere Vorgangsweise,
- relevante extrapulmonale Zufallsbefunde (z. B. schwerer Koronarkalk, schweres Emphysem) sowie
- eine zusammenfassende Einschätzung („summary of LCS findings and suggested management“),

erfasst werden.

Zur standardisierten Kategorisierung, Festlegung von Follow-up-Intervallen und Managementpfaden sollten internationale Leitlinien wie die der ESTI, Lung-RADS 1.1 (Lung Imaging Reporting and Data System), die British Thoracic Society Guidelines herangezogen werden sollen.

Die konsequente Anwendung volumetrischer Messungen und standardisierter Reporting-Systeme reduziert Überdiagnosen und unnötige invasive Eingriffe und ist Voraussetzung für die Vergleichbarkeit zwischen Zentren und über die Zeit. Die ESTI empfiehlt in diesem Zusammenhang ein systematisches Qualitätsmanagement mit enger Zusammenarbeit mit der medizinischen Physik sowie regelmäßiger Überprüfung von Dosis, Bildqualität, Scannerleistung und Leserperformance.

## Interdisziplinärer Patientenpfad

- Ein LDCT-Screening-Programm erfordert eine multidisziplinäre Struktur, um eine rasche und qualitätsgesicherte Abklärung suspekter Befunde zu gewährleisten [25]. Erstkontakt: Vor der Teilnahme erfolgen ein ärztliches Gespräch mit Prüfung der Eignung (Erstberatung) und eine Rauchstopp-Beratung. Diese Maßnahmen werden unter definierten Voraussetzungen von einer hausärztlichen, internistischen/lungenfachärztlichen oder betriebsmedizinischen Praxis durchgeführt.
- Radiologische Praxis oder Abteilung: Aufklärung über die Niedrigdosis-CT-Untersuchung sowie Durchführung und Befundung nach ESTI-Standards, weiteres Vorgehen, Follow-up-Planung, Qualitätssicherung gemäß der entsprechenden Leitlinie der ERS, ESR und ESTI.
- Pneumologische Abteilung: klinische Einschätzung, weiterführende Diagnostik, Management im Austausch im multidisziplinären Team mit Radiologie und Thoraxchirurgie und Feedback an Allgemeinmediziner:innen/niedergelassene Pneumolog:innen.
- Thoraxchirurgie (Beurteilung operabler Läsionen, kurative Therapieoptionen),
- sowie – je nach Befundlage – Pathologie und Onkologie.

Als Hochrisiko klassifizierte Befunde sollten in einem Kompetenzzentrum (z. B. Rundherdambulanz) vorgestellt und einer möglichen Diagnostik zugeführt werden. Strukturierte, pulmoradiologische Rundherdboards können additiv herangezogen werden. Eine interdisziplinäre Entscheidungsfindung stellt sicher, dass Patient:innen rasch und leitliniengerecht einer adäquaten Diagnostik und Therapie zugeführt werden, und trägt dazu bei, Überdiagnosen und unnötige invasive Eingriffe zu minimieren.

## Organisation und Infrastruktur

Ein nationales Lungenkrebs-Screening erfordert eine klar definierte, qualitätsgesicherte Organisationsstruktur, die sowohl diagnostische Sicherheit als auch effiziente Prozesse gewährleistet. Die Implementierung soll ausschließlich in zertifizierten Screening-Zentren erfolgen, die über standardisierte Abläufe, geeignete technische Ausstattung und spezifische Expertise verfügen. Solche Zentren müssen LDCT-Protokolle gemäß ESTI-/ESR-Standards verwenden, über moderne Mehrzeilen-CT-Systeme mit niedriger Strahlendosis verfügen und umfassende Qualitätsmanagementsysteme implementieren.

## Zertifizierte Screening-Zentren

Zentren, die LDCT-Screening anbieten, sollten anhand klar definierter Kriterien zertifiziert werden. Dazu zählen:

- standardisierte LDCT-Protokolle und volumetrische Analysetools,
- ausgewiesene Erfahrung in der thorakalen Radiologie,
- strukturiertes Reporting gemäß ESTI-Templates,
- Zugang zu interdisziplinären Abklärungsstrukturen,
- dokumentierte Teilnahme an Schulungen und Weiterbildungen,
- definierte Prozesse für Patient:innenführung, Follow-up und Nachverfolgung.

Die Zertifizierung stellt sicher, dass Befunde reproduzierbar, vergleichbar und leitliniengerecht erhoben und interpretiert werden und dass die Behandlungspfade zentral gesteuert werden können.

## Qualitätssicherung

Die Qualitätssicherung ist ein zentrales Element jedes Screening-Programms und umfasst mehrere Ebenen. Zentral müssen die erforderlichen Schulungskriterien, die erreichten Screening-Zahlen, die Korrektheit der Einschlusskriterien, die erreichte Stadienverteilung, der gleichförmige Zugang über alle sozioökonomischen Schichten und die Interventions‑/Outcome-Rate monitorisiert werden.

Ein wesentlicher Bestandteil der Qualitätssicherung ist die radiologische Befundung, die auf etablierten Leitlinien wie denen der ESTI oder Lung-RADS 1.1. Einheitliche Templates und eine strukturierte Befundung tragen dazu bei, interindividuelle Variabilität zu reduzieren und die diagnostische Präzision zu erhöhen.

Ein weiterer Kernbereich ist die kontinuierliche Schulung aller beteiligten Fachgruppen. Radiolog:innen, Pneumolog:innen und Thoraxchirurg:innen müssen regelmäßig Fortbildungen zu Bildinterpretation – einschließlich Volumetrie und Wachstumsratenanalyse –, zum Einsatz Screening-spezifischer Software, zur standardisierten Dokumentation sowie zur Risikostratifizierung und patientenzentrierten Kommunikation absolvieren.

Zunehmend setzen europäische Programme auf ein Single-Reading-Modell durch qualifizierte Radiolog:innen, das durch KI-basierte Assistenzsysteme ergänzt wird. Diese Systeme unterstützen die automatische Detektion von Rundherden, die volumetrische Analyse, den Vergleich mit Vorbefunden sowie die Ableitung geeigneter Follow-up-Empfehlungen nach ESTI-, Lung-RADS- oder europäischen Standards. Dadurch werden sowohl diagnostische Konsistenz als auch Effizienz gesteigert, und gleichzeitig wird der wachsende Arbeitsaufwand abgefedert.

Regelmäßige interne und externe Audits bilden eine weitere Säule der Qualitätssicherung. Sie überprüfen die Einhaltung definierter Prozessstandards, kontrollieren Bildqualitätsparameter wie CTDIvol, Dosisreduktion und Rekonstruktionstechniken und evaluieren Abklärungs- und Biopsieraten sowie relevante Outcome-Daten. Darüber hinaus dienen sie der Überwachung von Stadienshift, Compliance und Intervallkarzinomen. Die Auswertung der Auditdaten sollte möglichst zentral erfolgen – idealerweise über eine nationale Koordinierungsstelle oder ein Screening-Register –, um eine einheitliche, qualitätsgesicherte Steuerung des Programms zu gewährleisten.

## IT-Infrastruktur

Ein effektives und sicheres Screening-Programm benötigt eine robuste digitale Infrastruktur, die folgende Komponenten umfasst:

- zentrales Bildarchiv (Picture Archiving and Communication System/PACS) mit standardisierten LDCT-Protokollen,
- automatisierte Übertragungen zur volumetrischen Analyse,
- zentrales Screening-Register, in dem Daten zu Risikoprofil, Befunden, Follow-up und Outcomes gespeichert werden,
- Integration von Künstlicher Intelligenz-Tools zur Prozessunterstützung,
- Schnittstellen zur Primärversorgung und zu Rundherdboards,
- Einladungssysteme (Recall, Reminder),
- datenschutzkonforme Speicherung und Pseudonymisierung.

Ein nationales Register ist essenziell, um Qualitätssicherung, Gesundheitsökonomie, Outcomes und laufende Programmevaluierung zu gewährleisten.

## Verpflichtende Lungenrundherdboards

Zur strukturierten und leitliniengerechten Abklärung suspekter Befunde sind verpflichtende Lungenrundherdboards erforderlich. Diese multidisziplinären Boards bestehen in der Regel aus Radiologie, Pneumologie, Thoraxchirurgie, Onkologie (bei Bedarf), Pathologie, Strahlentherapie (je nach Zentrum).

Sie gewährleisten eine gemeinsame Entscheidungsfindung bei unklaren oder potenziell malignen Befunden, eine Definition der weiteren diagnostischen Schritte, Minimierung unnötiger invasiver Eingriffe, rasche Zuweisung in kurative Therapiepfade und eine kontinuierliche Qualitätssicherung durch systematisches Feedback und Outcome-Analyse.

Diese Boards bilden das Rückgrat der diagnostischen Sicherheit und sind zentraler Bestandteil aller erfolgreichen europäischen Screening-Modelle.

## Begleitmaßnahmen

Ein wirksames nationales Lungenkarzinom-Screening-Programm umfasst mehr als die reine Durchführung von LDCT-Untersuchungen. Entscheidend für den langfristigen Erfolg ist die Implementierung umfassender Begleitmaßnahmen, die sowohl gesundheitspolitische als auch verhaltenspräventive Aspekte adressieren. Diese Maßnahmen tragen wesentlich zur Erhöhung der Teilnahmequote, zur Verbesserung der Versorgungsqualität und zur Reduktion des individuellen wie gesellschaftlichen Lungenkrebsrisikos bei.

## Raucher:innenentwöhnungsprogramme

Zentrale Säule eines jeden Screening-Programms ist die systematische Integration der Raucher:innenentwöhnung. Europäische Fachgesellschaften (ERS/ESR) und die EU-Kommission betonen, dass Tabakentwöhnung verpflichtend und strukturiert in das Screening eingebettet sein muss und allen Teilnehmern eine entsprechende Entwöhnung angeboten werden sollte. Dies umfasst:

- Beratung im Rahmen des Screenings,
- standardisierte „Brief Counselling“-Elemente (5A/5R),
- Weiterleitung zu Entwöhnungsprogrammen (telefonisch, digital, ambulant),
- Zugang zu evidenzbasierten Interventionen (Nikotinersatz, medikamentöse Therapien),
- Dokumentation und Follow-up im Screening-Register.

Die Kombination von LDCT-Screening und Raucher:innenentwöhnung führt nicht nur zu einer Reduktion der Lungenkrebssterblichkeit, sondern senkt zusätzlich das Risiko für COPD, kardiovaskuläre Erkrankungen und andere tabakassoziierte Morbiditäten [14, 15, 17].

## Öffentlichkeitsarbeit und Aufklärung

Ein breit angelegtes Screening-Programm erfordert eine strukturierte Öffentlichkeitsarbeit, die sachlich über Nutzen und Risiken informiert und die Teilnahmebereitschaft erhöht. Wesentliche Elemente sind:

- Informationskampagnen in der Allgemeinbevölkerung und bei Hochrisikogruppen,
- transparente Darstellung von Nutzen (Mortalitätsreduktion, frühe Diagnose) und Risiken (Strahlung, falsch positive Befunde, Überdiagnosen),
- zielgruppengerechte Ansprache über Primärversorgung, Arbeitsmediziner:innen, Apotheken und kommunale Einrichtungen,
- Bereitstellung niedrigschwelliger Informationsmaterialien (Website, Broschüren, digitale Tools),
- Zusammenarbeit mit Patient:innenorganisationen und Fachgesellschaften.

Eine klare und vertrauenswürdige Kommunikation ist entscheidend, um Missverständnisse zu vermeiden, Fehlinformationen entgegenzuwirken und die Akzeptanz in der Bevölkerung zu stärken.

## Fortbildung für Ärzt:innen

Für eine erfolgreiche Implementierung ist die kontinuierliche Fortbildung aller am Screening beteiligten Fachgruppen unerlässlich. Dazu gehören niedergelassene Ärzt:innen der Allgemeinmedizin und Pneumologie, Arbeitsmediziner:innen, Radiolog:innen und Radiologietechnolog:innen, Thoraxchirurg:innen und Case Manager:innen. Die Fortbildungen umfassen Themen wie Risikostratifizierung und die Definition von Ein- und Ausschlusskriterien, die Interpretation von Screening-Befunden, die verständliche Kommunikation von Nutzen und Risiken, standardisierte Abklärungsalgorithmen sowie die strukturierte Organisation der Nachsorge. Regelmäßige Schulungen tragen wesentlich dazu bei, eine konsistente und qualitätsgesicherte Versorgung über die gesamte Screening-Kette hinweg sicherzustellen. Eine Basis für die Fortbildung von Radiologen könnte hier das Zertifizierungsprogramm der ESTI sein.

## Nutzen-Risiko-Analyse und AIHTA-Review

Screening-Teilnehmer:innen müssen umfassend über Risiken und Vorteile der Lungenkrebsvorsorge aufgeklärt werden. Diese Informationen sollten niederschwellig zur Verfügung stehen. Ziel ist, dass für die einzelne Person der zu erwartende Nutzen des Screenings – Senkung der lungenkrebsbedingten Mortalität und Zugewinn an Lebensjahren – die Risiken des Screenings und der resultierenden Abklärungsmaßnahmen übersteigt. Dies ist am ehesten dann zu erwarten, wenn die Person ein entsprechend hohes Erkrankungsrisiko aufweist und gleichzeitig gesund genug ist, um von dem Zugewinn zu profitieren.

Die aktuellen Leitlinien der American Cancer Society (ACS) empfehlen daher kein LDCT-Screening über dem Alter von 80 Jahren oder bei Vorliegen relevanter Komorbiditäten [30]. Eine exakte Definition der Risikopopulation ist daher ein zentraler Punkt des LDCT-Screenings, ebenso wie die Festlegung eines optimalen Screening-Intervalls.

Für Deutschland hat das Institut für Qualität und Wirtschaftlichkeit im Gesundheitswesen (IQWiG) bereits 2020 in einer Nutzenbewertung festgestellt, dass für aktive und ehemalige Raucher:innen der Nutzen eines LDCT-Lungenkrebs-Screenings den potenziellen Schaden überwiegt [31, 32].

Das AIHTA kommt – auf Basis österreichischer und internationaler Daten – zu einer klar positiven Nutzen-Risiko-Bilanz, insbesondere in einer hochrisikoadaptierten Zielgruppe [33].

Als mögliche Risiken eines Lungenkarzinom-Screenings werden insbesondere falsch positive Befunde, Überdiagnosen/Übertherapien, kumulative Strahlendosis und psychologische Belastung angeführt. Die psychische Belastung, die ein positives Screening-Ergebnis mit sich bringen kann, ist noch nicht umfassend geklärt; v. a. angesichts potentieller falsch positiver Befunde muss diesem Aspekt besondere Beachtung geschenkt werden [34]. Eine sorgfältige Aufklärung vor Durchführung der Screening-Untersuchung ist daher essenziell.

## Kosteneffektivität eines LDCT-Screenings in Österreich

Eine zentrale Frage bei der Implementierung eines nationalen Lungenkrebs-Screenings ist dessen Kosteneffektivität. Diese hängt wesentlich von der Definition der Screening-Population, der Teilnahmequote sowie der weiteren Abklärung positiver Screening-Befunde ab.

In Europa betragen die durchschnittlichen Behandlungskosten pro Person bei Lungenkrebs im Frühstadium rund 16.291 €, im fortgeschrittenen Stadium etwa 22.175 € [35, 36]. Ein durch ein nationales Lungenkrebs-Screening bewirkter Stadienshift mit einer vermehrten Diagnose in frühen Tumorstadien könnte daher zu einer Reduktion krankheitsbedingter Folgekosten beitragen.

Für Österreich liegt eine gesundheitsökonomische Analyse vor [37], welche die Kosteneffektivität eines LDCT-basierten Lungenkrebs-Screenings eindeutig belegt. Bewertet wurden der Zugewinn an Lebensjahren, qualitätsadjustierte Lebensjahre (QALYs), die zusätzlichen Kosten für Screening, Diagnostik und Therapie sowie

- die inkrementelle Kosten-Effektivitäts-Ratio (ICER) pro gewonnenem QALY.

Die Screening-Population wurde analog zu den Einschlusskriterien der NELSON-Studie definiert. Insgesamt zeigt die Analyse, dass ein strukturiertes Lungenkrebs-Screening in Österreich kosteneffizient ist, mit einer ICER von 24.627 € pro QALY [37].

Daraus ergäbe sich für Österreich eine Screening-Population von rund 300.000 Personen; bei einer angenommenen Teilnahmequote von 50 % entspricht dies etwa 150.000 Personen. Rekrutierungskosten werden mit 15,7 Mio. €, Screening-Kosten mit 944,9 Mio. € berechnet. Zudem würden durch die Implementierung des Screenings rund 35,6 Mio. € für diagnostische Maßnahmen und 385,6 Mio. € an zusätzlichen therapeutischen Kosten entstehen.

Dennoch konnte gezeigt werden, dass es aufgrund eines relevanten Stadienshifts – also einer vermehrten Diagnose in frühen Tumorstadien – zu einer Reduktion der Therapiekosten im Stadium IV kommt.

Neuere gesundheitsökonomische Modellierungsstudien zeigen, dass die Kosteneffektivität von Lungenkrebs-Screening-Programmen durch die zunehmende Verfügbarkeit wirksamer, jedoch kostenintensiver Therapien für fortgeschrittene Erkrankungsstadien weiter verbessert werden kann [38, 39]. Durch die frühere Diagnosestellung und die Verschiebung hin zu kurativ behandelbaren Tumorstadien können nicht nur zusätzliche Lebensjahre gewonnen, sondern potenziell auch Kosten fortgeschrittener Krankheitsverläufe reduziert werden [38, 39]. Diese Ergebnisse werden durch eine aktuelle systematische Übersichtsarbeit sowie mehrere Modellierungsstudien aus Deutschland, Großbritannien, Kanada und den USA bestätigt [40–45].

Die Kosteneffizienz kann zusätzlich durch die Integration von Raucher:innenentwöhnungsprogrammen verbessert werden, da sowohl die Entwöhnungsraten, als auch die Bereitschaft zur Teilnahme an entsprechenden Entwöhnungsprogrammen im Rahmen von Screening-Programmen nachweislich erhöht sind [46, 47].

Ein weiterer Faktor, der die Kosteneffektivität steigert, ist eine hohe Screening-Adhärenz. Im Rahmen eines strukturierten Programms sollten daher Maßnahmen gesetzt werden, die regelmäßige Teilnahme fördern und langfristig sichern.

## Herausforderungen und Limitationen

Trotz klarer Evidenz für die Mortalitätsreduktion durch LDCT-Screening bestehen relevante organisatorische und medizinische Herausforderungen, die bereits in der Planungsphase adressiert werden müssen.

## Rekrutierung: Einbindung relevanter Partner:innen

Die erfolgreiche Identifikation und Rekrutierung der Risikopopulation erfordert eine breite Vernetzung verschiedener Sektoren des Gesundheitswesens. Eingebunden werden sollten u. a.:

- Allgemeinmediziner:innen: primäre Anlaufstelle, zentrale Rolle bei Risikostratifizierung, Erstberatung und Motivation, Follow-up,
- Radiolog:innen: Untersuchung, Befunderstellung, Vorgabe weiterer Maßnahmen, strukturierte Weiterleitung, Terminvergabe von Follow-up Untersuchungen,
- Pneumolog:innen: Identifikation von Hochrisikopatient:innen mit COPD und mit langjähriger Raucher:innenanamnese,
- Kardiolog:innen: Betreuung von Patient:innen mit Kornonarer Herzkrankheit (KHK) und hoher Rauchbelastung,
- arbeitsmedizinische Dienste: Erreichbarkeit von Personen mittleren Alters mit hoher beruflicher Belastung und erhöhter Rauchprävalenz.

Ein mögliches digital unterstütztes Einladungs- und Reminder-System kann die Teilnahmequote weiter erhöhen und eine konsistente Nachverfolgung gewährleisten.

## Umgang mit Komorbiditäten und Zusatzbefunden

Die Zielgruppe des LDCT-Screenings weist häufig relevante Komorbiditäten wie koronare Herzkrankheit, Emphysem oder interstitielle Lungenerkrankungen auf [48]. Daher braucht es eine klare Grundlage für den Umgang mit Zufallsbefunden einschließlich deren Kommunikation und Dokumentation. Darüber hinaus sollen strukturierte Abklärungspfade für Patient:innen mit ausgeprägten Komorbiditäten etabliert werden, um Überdiagnosen, Fehlinterpretationen und unnötige invasive Eingriffe zu reduzieren.

## Logistische Umsetzung und Ressourcenbedarf

Unter der Annahme einer Teilnahme von 50 % der Risikopopulation ergibt sich ein Bedarf an strukturierter Versorgung. Dazu gehört eine österreichweite, patientengerechte Standortverteilung mit LDCT-fähigen Zentren in den Bundesländern sowie definierten Partnerspitälern für multidisziplinäre Boards. Gleichzeitig ist der Personalbedarf, insbesondere für Radiolog:innen zur Befundung und Qualitätssicherung, Radiologietechnolog:innen für den Durchsatz an LDCT-Untersuchungen, Pneumolog:innen und Thoraxchirurg:innen für weiterführende Abklärungen, Case-Manager:innen für die Koordination der Patient:innenwege sowie Fachkräfte für IT und Datenmanagement, zu kalkulieren.

Auch diagnostische und interventionelle Kapazitäten müssen vorhanden sein, dazu gehören unter anderem ausreichende CT-Termine für Verlaufsuntersuchungen und bildgesteuerte Biopsien, modern ausgestattete Bronchoskopieeinheiten inklusive Endobronchialem Ultraschall (EBUS) und Navigationssystemen, thoraxchirurgische Ressourcen (einschließlich minimalinvasiver Verfahren) sowie Strukturen für Erstabklärungen. Insgesamt muss die Infrastruktur so ausgelegt sein, dass ein zeitnaher und effizienter Abklärungsprozess – idealerweise innerhalb von 4 Wochen – gewährleistet werden kann.

## Sicherstellung der regelmäßigen Teilnahme (Adhärenz)

Ein Screening ist nur dann wirksam, wenn die Teilnehmer:innen regelmäßig wiederkehren. Dafür sind effiziente Einladungssysteme mit Recall-Funktionen ebenso notwendig, wie mögliche digitale Patient:innenportale, die Terminerinnerungen und eine transparente Befundkommunikation ermöglichen. Ergänzend braucht es eine strukturierte Beratung, die die Bedeutung einer kontinuierlichen Teilnahme vermittelt, sowie Rückmeldungen zur Wirksamkeit des Programms, etwa durch Beispiele für entdeckte Frühstadien. Eine starke Einbindung von Allgemeinmediziner:innen und niedrigschwellige Zugangsmöglichkeiten – etwa flexible CT-Termine und wohnortnahe Zentren – sind ebenfalls zentrale Voraussetzungen. Erfahrungen aus NLST, NELSON und britischen Programmen zeigen klar, dass die Adhärenz maßgeblich für den Mortalitätsbenefit ist.

## Forderungen der Fachgesellschaften

Die Österreichische Röntgengesellschaft (ÖRG), die Österreichische Gesellschaft für Pneumologie (ÖGP), die Österreichischen Gesellschaft für Allgemeinmedizin und Familienmedizin (ÖGAM) und die Österreichische Gesellschaft für Thoraxchirurgie (ÖGTC) sprechen sich gemeinsam für folgende Schritte aus:

1. *Einführung eines nationalen Lungenkarzinom-Screenings* in Österreich mit strukturiertem, qualitätsgesichertem Patient:innenpfad,
2. *Definition zielgerichteter Screening-Kriterien* gemäß evidenzbasierten Leitlinien und Risikomodellen,
3. *Harmonisierung von Plattformen für Datenmanagement* mit einem nationalen Register (z. B. Integration in ELGA),
4. *Start mit Pilotphasen mit zentraler Evaluierung und schrittweiser Ausrollung *mit anschließender landesweiter Implementierung,
5. *Sicherstellung der interdisziplinären Ressourcen* in Allgemeinmedizin, Radiologie, Pneumologie, Thoraxchirurgie,
6. *verpflichtendes Angebot einer hochwertigen Raucher:innenentwöhnung* als integraler Bestandteil des Screening-Programms.

## Data Availability

Alle dieser Arbeit zugrunde liegenden Daten sind in diesem Artikel enthalten.
